# Joint Analysis of Cardiovascular Control and Shear Wave Elastography to Determine Carotid Plaque Vulnerability †

**DOI:** 10.3390/jcm14020648

**Published:** 2025-01-20

**Authors:** Vlasta Bari, Beatrice Cairo, Francesca Gelpi, Fabiana Fancoli, Nicoletta Curcio, Giulia Matrone, Paolo Righini, Giovanni Nano, Alberto Porta, Daniela Mazzaccaro

**Affiliations:** 1 Department of Biomedical Sciences for Health, University of Milan, 20133 Milan, Italy; beatrice.cairo@unimi.it (B.C.); giovanni.nano@unimi.it (G.N.); alberto.porta@unimi.it (A.P.); 2 Department of Cardiothoracic, Vascular Anaesthesia and Intensive Care, IRCCS Policlinico San Donato, 20097 San Donato Milanese, Italy; francesca.gelpi@grupposandonato.it; 3 Operative Unit of Vascular Surgery, IRCCS Policlinico San Donato, 20097 San Donato Milanese, Italy; fabiana.fancoli@grupposandondato.it (F.F.); paolo.righini@grupposandonato.it (P.R.); daniela.mazzaccaro@grupposandonato.it (D.M.); 4 3D and Computer Simulation Laboratory, IRCCS Policlinico San Donato, 20097 San Donato Milanese, Italy; nicoletta.curcio@grupposandonato.it; 5 Department of Electrical, Computer and Biomedical Engineering, University of Pavia, 27100 Pavia, Italy; giulia.matrone@unipv.it

**Keywords:** baroreflex, autonomic nervous system, elastography, Young’s modulus, carotid artery stenosis, carotid plaque vulnerability

## Abstract

**Background/Objectives**: Carotid artery stenosis (CAS) is one of the main causes of stroke, and the vulnerability of plaque has been proved to be a determinant. A joint analysis of shear wave elastography, a radiofrequency echo-based wall tracking technique for arterial stiffness evaluation, and of autonomic and baroreflex function is proposed to noninvasively, preoperatively assess plaque vulnerability in asymptomatic CAS patients scheduled for carotid endarterectomy. **Methods**: Elastographic markers of arterial stiffness were derived preoperatively in 78 CAS patients (age: 74.2 + 7.7 years, 27 females). Autonomic and baroreflex markers were also assessed by means of an analysis of the beat-to-beat fluctuations in heart period and systolic arterial pressure, derived at rest in supine position (REST) and during active standing. Postoperative analysis identified 36 patients with vulnerable plaque (VULN) and 42 with stable plaque (STABLE). **Results**: Baroreflex sensitivity (BRS) at a respiratory rate decreased during STAND only in VULN patients, being much higher at REST compared to STABLE levels. Autonomic indexes were not helpful in separating experimental conditions and/or populations. The Young’s modulus (YM) of the plaque was lower in the VULN group than in the STABLE one. Cardiovascular control and elastographic markers were significantly correlated only in VULN patients. A multivariate logistic regression model built combining YM and BRS at the respiratory rate improved the prediction of plaque vulnerability, reporting an area under the ROC curve of 0.694. **Conclusions**: Noninvasive techniques assessing shear wave elastography and baroreflex control could contribute to the early detection of plaque vulnerability in patients with asymptomatic CAS.

## 1. Introduction

Carotid artery stenosis (CAS) as a consequence of atherosclerotic plaque presence is one of the main causes leading to stroke [1,2]. Symptomatic CAS is usually treated with carotid endarterectomy (CEA) or stenting and with medical therapies. The management of asymptomatic CAS is instead more uncertain, since trials reporting the effectiveness of surgery in these patients date back 20 years ago or more, while recent improvements suggest that medical therapy could be as beneficial as CEA or stenting to prevent stroke [3,4]. In particular, the presence of neurologic symptoms such as stroke or transient ischemic attack in a patient with symptomatic carotid stenosis contributes to the vulnerability of the patient, since it has been extensively reported in the literature that symptomatic patients have a higher risk of stroke recurrence. In such patients, the indication of carotid revascularization is straightforward to prevent any further possible neurologic complication. In asymptomatic patients, however, assuming the same degree of stenosis for all patients (i.e., >70%), vulnerability can be defined only via the histologic analysis of the plaque [5].

As a matter of fact, it has been shown that the risk of stroke in asymptomatic CAS is more related to the structure of the atherosclerotic plaque, with some plaques being more vulnerable and prone to rupture than more stable ones [6]. The vulnerability of the plaque would thus be directly linked to the occurrence of stroke [7], but the gold standard technique used to assess plaque vulnerability has so far been considered postoperative histological analysis [8]. As a consequence, identifying vulnerable plaque at an early stage has become more and more important, especially by means of noninvasive techniques [5,9].

Markers of arterial stiffness as assessed via pulse wave velocity have been found to be useful to assess atherosclerosis [10], while other works have identified the ability of imaging, such as computed tomography angiography, magnetic resonance scans, or 2D duplex ultrasound, to identify the characteristics of atherosclerotic plaques [11,12]. However, there is a need for more advanced technologies to also assess the characteristics of plaque vulnerability.

In this direction, ultrasound elastography and, in particular, strain and shear wave elastography, have been suggested as able to identify the mechanical properties of the vascular system, more specifically the elastic properties of the carotid walls, according to the paradigm that higher-stiffness regions are linked to the presence of calcifications, while lower-stiffness regions are linked to soft or hemorrhagic tissue and hence to a more vulnerable plaque [13,14]. In this sense, shear wave elastography (SWE) has been proposed as a noninvasive imaging technique able to identify carotid plaque elasticity [15] and vulnerability, as compared to traditional histology [16]. A preliminary study by our group has also shown that point SWE (pSWE) allowed for the discrimination of patients with vulnerable plaque [17].

Autonomic nervous system function and cardiovascular control, which can be noninvasively assessed from the study of spontaneous fluctuation in heart period (HP) and systolic arterial pressure (SAP), have been found to be depressed in cases of increased cardiovascular risk [18,19]. Several studies have linked the state of autonomic nervous system control to the incidence of cerebrovascular adverse events, like microinfarct or stroke, after a surgical procedure; either vascular or cardiac surgery and their likelihood is also dependent on the age of the patients. As an example, a greater blood pressure variability, usually associated with a dysfunction in baroreflex control, has been associated with a more rapid cognitive decline and a higher likelihood of silent stroke, especially in the elderly [20,21]. Furthermore, reduced autonomic control has been linked to stroke severity [22,23] and to an increased risk of mortality after cerebral ischemia [24].

Such markers have also been proposed to characterize the state of patients with CAS and the surgical impact of CEA [25,26], finding that cardiovascular control was compromised in patients undergoing CEA [26]. As to the relationship of autonomic function with carotid plaque, it has been reported that the presence of carotid atherosclerotic plaque was associated with a reduced heart rate variability [27], but how cardiovascular control can be linked to plaque vulnerability has not been investigated so far.

We hypothesize that the investigation of autonomic control and especially cardiac baroreflex, the mechanism adjusting variations in HP in response to variations in SAP [28,29], could help in differentiating patients with vulnerable plaque and that the addition of such analysis to ultrasound pSWE, which has already been proved useful in identifying patients with vulnerable plaque, and to arterial stiffness evaluation could further improve risk stratification.

Hence, the aim of this research was to study cardiovascular control by means of a joint analysis of variability series of HP and SAP during a postural challenge and by the elastographic characteristics of the carotid plaque, as derived from pSWE and from ultrasound radiofrequency (RF) echo-based wall tracking in the same cohort of patients with asymptomatic CAS scheduled for CEA. The ability of such markers to predict the vulnerability of the plaque, as defined by the postoperative clinical observation [8], was then assessed via a receiver operating characteristic (ROC) curve analysis. The preliminary results have been presented at the Computing in Cardiology Conference 2024.

## 2. Materials and Methods

### 2.1. Experimental Protocol

One hundred patients with asymptomatic CAS of 70–99% scheduled for CEA were enrolled at the Department of Vascular Surgery of IRCCS Policlinico San Donato, San Donato Milanese, Milan, Italy. The study adhered to the Helsinki declaration for studies involving humans and was approved by the San Raffaele Hospital ethical committee (approval no. 110/int/2019, date 20 June 2019) and registered on ClinicalTrial.gov (Identifier: NCT05566080). Patients signed a written informed consent prior to participation.

Exclusion criteria were age under 18, non-sinus rhythm, presence of medical conditions for which survival expectancy was below 12 months, unstable or uncontrolled medical conditions (NYHA class III or IV heart failure or angina pectoris, previous cardiac surgery within 30 days, left ventricular ejection fraction below 30%, severe coronary artery disease with indication for surgery), medical history of stroke, or transient ischemic attack in the previous 6 months. After the exclusion of 22 patients for different reasons (bad signal recordings, missing data, previously unknown arrhythmias), 78 patients (age: 74.2 + 7.7 years, 27 females) were admitted to the analysis. Acquisition sessions were performed before surgery. After surgery, patients were divided in two classes: those with a vulnerable plaque (VULN) and those with a non-vulnerable plaque (STABLE) according to the presence of at least one of the following characteristics: ulceration, ruptured, or thin (<65 µm) fibrous cap, necrotic or lipidic core for more than 25% of the total area, large intraplaque hemorrhage, infiltration of inflammatory cells, neovascularization of the cap [8]. Hence, 36 patients were assigned to the VULN group and 42 to the STABLE one. The clinical features of the two groups are depicted in Table 1.

### 2.2. Elastographic Markers Assessment

As described in [17], acquisition of pSWE was performed via an Esaote MyLab ultrasound system (EsaoteTM, Genova, Italy), with patients lying in supine position and with a slight neck extension in the region of the carotid artery. A probe working at 7.5 MHz and the Q-Elaxto software package (EsaoteTM, Genova, Italy) were used, while the quality arterial stiffness (QAS) software was used to derive arterial stiffness-related parameters through RF echo-based wall tracking. To perform acquisitions, the probe was placed along a longitudinal axis passing along the distal part of the carotid artery just below the atherosclerotic plaque. The QAS algorithm allowed us to automatically derive real-time measurements of the diameter change in the vessel between systolic and diastolic phases. The carotid pressure waveform was derived from the brachial pressure and cross-sectional area of the vessels during the cardiac cycle. From the ultrasound images, QAS allowed us to derive different markers of arterial stiffness from the changes in vessel area in relation to the local pressure during diastolic and systolic phases: (i) distensibility coefficient (DC), expressed in 1·kPa^−1^ and defined as DC = ∆A/(A·Δp), where ΔA is the change in area during systole, A is the diastolic area of the vessel, and Δp is the local pulse pressure; (ii) compliance coefficient (CC), expressed in mm^2^·kPa^−1^ and defined as DC = ΔA/Δp; (iii) index of alpha stiffness (α index), representing the elastic coefficient of the vessel calculated as α = A·ln(SAP/DAP)/ΔA, where SAP and DAP are, respectively, systolic and diastolic pressure, and ln is the natural logarithm; (iv) index of beta stiffness (β index), that is the elastic coefficient normalized by the vessel diameter, calculated as β = D·ln(SAP/DAP)/ΔD, where D is the diastolic diameter and ΔD is the change in diameter during systole; (v) pulse wave velocity (PWV), calculated in m∙s^−1^ as PWV = 1/$\sqrt{\left(\rho \cdot A-\Delta p/\Delta A\right)}$, where ρ is the blood density and Δp is the local pulse pressure; (vi) augmentation index (AIx), assessed as AIx = [LocSAP−P(T1)/(LocSAP −LocDAP)] × 100, where LocSAP and LocDAP are local SAP and DAP and P(T1) is the pressure at the inflection points. Finally, the pSWE allowed us to compute the Young’s elastic modulus (YM) of the plaque, expressed in kPa, representing the arterial stiffness [17,30].

### 2.3. Variability Series Extraction

Electrocardiogram (ECG) lead II (BioAmp, Adinstruments, Sydney, Australia) and noninvasive arterial pressure (AP) as derived from volume-clamp photopletysmography (CNAP, CNSystems, Graz, Austria) were recorded for 10 min with patients lying in supine position at REST and for 10 min during active standing (STAND). Signals were recorded at a sampling frequency of 400 Hz and synchronized with a polygraph (PowerLab, Adinstruments, Sydney, Australia). From the signals, we extracted the beat-to-beat time series of HP, taken as the time distance between two consecutive R-wave peaks of the ECG detected with minimum jitters via parabolic interpolation, and of SAP, taken as the maximum of the AP signal within the HP. Respiration (RESP) was derived from the ECG amplitude modulations. Series of 256 consecutive beats for HP, SAP, and RESP were extracted during both REST and STAND and checked by a trained operator. Ectopic beats and misdetections were corrected by means of linear interpolation between the closest reliable values.

Mean values were extracted from the series, labeled as μ_HP_, μ_SAP_, and expressed in ms and mmHg. Then, after linear detrending of the series, variance of the time series was computed and, respectively, labeled as σ^2^_HP_ and σ^2^_SAP_ and expressed in ms^2^ and mmHg^2^.

**Figure 1:**
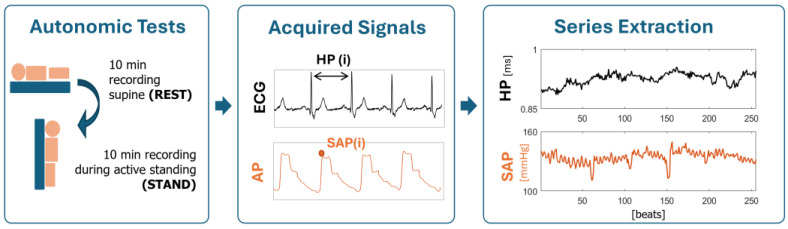
Experimental setup of cardiovascular signals acquisition. ECG = electrocardiogram; AP = arterial pressure; HP = heart period; SAP = systolic arterial pressure; i= ith beat; REST = at rest in supine position; STAND = during active standing.

Figure 1 shows the experimental setup.

### 2.4. Cardiovascular Control Markers Extraction

Consequently, the power spectral density of the series was computed to obtain univariate cardiovascular control spectral markers. The description of the dynamics was performed via an autoregressive model whose coefficients were identified via least squares method solved via Levinson–Durbin recursion. The number of coefficients was optimized via Akaike information criterion in the range 10–14 [31,32]. The power spectral density was factorized in spectral components. The area under each spectral component was computed via the residue theorem and the sum of the areas was equal to the variance of the series. Each component was labeled as low (LF, 0.04–0.15 Hz) or high (HF, 0.15–0.4 Hz) frequency according to the value of its central frequency [33]. The power of HP in the HF band in absolute units (HF_HP_, expressed in ms^2^) was associated to the vagal modulation directed to the ventricles [34], while the power of SAP in the LF band in absolute units (LFS_AP_, expressed in mmHg^2^) was depicted as a marker of sympathetic modulation directed to the vessels [19]. The respiratory frequency f_RESP_ was computed from the RESP series as the frequency at which the maximum in the HF band occurred.

Bivariate cross-spectral AR analysis was then used to derive baroreflex markers. Cross-spectral density function was computed from the parameters of the autoregressive bivariate model identified by solving the least squares problem via Cholesky decomposition, with the model order fixed to 10 [35]. The same method provided an estimation of the power spectral densities. The transfer function gain (TFG) was estimated as the ratio of the modulus of the cross-spectral density function from SAP to HP divided by the power spectral density of the input (e.g., SAP), while the squared coherence (K^2^) was computed as the ratio of the square modulus of the cross-spectral density function to the product of the power spectral densities of SAP and HP. K^2^ ranged between 0 and 1, where 0 means null and 1 full coupling. The TFG was sampled at the maximum of K^2^ function in the LF and HF bands, and this value, expressed in ms·mmHg^−1^, was taken as a marker of baroreflex sensitivity, labeled, respectively, as BRS_LF_ and BRS_HF_ [36]. The K^2^ was also sampled at its maximum in the LF and HF bands, and these markers, indicated as K^2^_LF_ and K^2^_HF_, were taken as representative of the coupling between HP and SAP. The phase of the cross-spectral density from SAP to HP in the LF and HF bands, expressed in rad and labeled as Ph_LF_ and Ph_HF_, was used as representative of the phase shift from SAP to HP, where negative values indicate that HP lags behind SAP.

### 2.5. Statistical Analysis

Clinical and demographic data were compared between VULN and STABLE patients via t test, or Mann–Whitney rank sum test when appropriate in the case of continuous variables, and chi-square test, in the case of categorical variables. We utilized the t test or Mann–Whitney rank sum test when appropriate to compare the elastographic markers between the two groups. Two-way repeated measures analysis of variance (ANOVA) with one factor repetition was used to compute differences between the patient populations (VULN and STABLE) and experimental conditions (REST and STAND) of cardiovascular variability markers. Holm–Sidak test was used to assess multiple comparisons. Pearson’s correlation coefficient was used to compute the relationship between elastographic and cardiovascular variability markers in STABLE and VULN patients. Analysis was carried out separately at REST and during STAND. The markers reporting a statistical difference between VULN and STABLE with *p* < 0.01 entered a multivariate logistic regression model. The regression coefficient, odds ratio, 95% confidence interval (CI), and type I error probability *p* of the multivariate regression model were computed. The area under the ROC curve (AUC) was assessed. The Youden index criterion was used to determine the best combination in terms of specificity and sensitivity and to assess the negative and positive predictive value (NPV and PPV).

## 3. Results

Table 1 depicts the clinical and demographic variables in STABLE and VULN patients. Remarkably, no differences were observed between the two populations.

Table 2 reports the results of elastographic markers assessed in the STABLE and VULN patients. Remarkably, only the YM of the plaque was significantly different between groups, being higher in STABLE plaque patients.

Table 3 shows markers of autonomic and cardiovascular control in the STABLE and VULN patients, assessed at REST and during STAND. µ_HP_ decreased at STAND as an effect of the postural challenge only in VULN patients. BRS_HF_ decreased during STAND in VULN patients with respect to REST and, at REST, resulted higher in VULN than in the STABLE patients. Remarkably, all the other indexes were not different between either group or condition.

Table 4 shows the results of the correlation between elastographic and autonomic and cardiovascular control markers assessed in STABLE patients. The majority of the autonomic and cardiovascular markers were not correlated with the elastographic markers, with the sole exception of the significant positive correlation of α_LF_ with AIx at REST.

Table 5 has the same structure as Table 4 but it refers to VULN patients. In this case, the number of significant correlations was larger. µ_HP,_ LF_SAP_ at REST, and µ_HP_, µ_SAP_, and σ^2^_SAP_ during STAND were positively correlated with the plaque’s YM. σ^2^_SAP_ during REST was negatively correlated with DC and positively correlated with the PWV. The HF_HP_ during STAND was positively correlated with the markers related to the artery stiffness: α index, β index, and PWV. Finally, all the elastographic, autonomic, and cardiovascular control markers in Table 2 and Table 3, taken separately at REST and during STAND, were compared between STABLE and VULN patients. The markers reporting a significance of the difference at the univariate level lower than 0.1 were selected to be entered in the following analyses. The chosen markers were the YM of the plaque and BRS_HF_ at REST.

**Figure 2:**
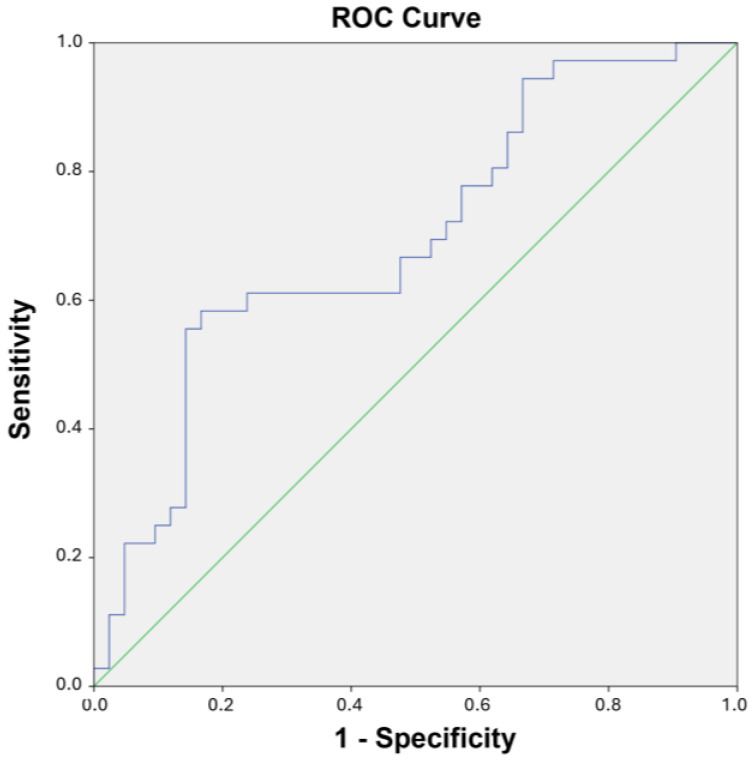
ROC curve related to the multivariate logistic regression model built with YM and BRS_HF_ evaluated at REST (blue). The green line represents the diagonal.

A logistic regression analysis was performed to test the ability of plaque’s YM and BRS_HF_ to predict the vulnerability of the plaque. The results of such analysis are presented in Table 6 in terms of odds ratio, 95% confidence interval (CI), *p*-value, and AUC. YM was significantly associated with the vulnerability of the plaque, reporting a *p*-value of 0.043 and an AUC of 0.625, while BRS_HF_ was less associated, reporting a *p*-value of 0.153 and an AUC of 0.616. The indexes then entered a multivariate logistic regression model, which reported an AUC equal to 0.694. The model was evaluated via the Youden Index criterion, reporting a sensitivity of 0.583 and a specificity of 0.833. The PPV was 80.3% and the NPV 63.2%. The ROC curve of the multivariate logistic model is shown in Figure 2.

## 4. Discussion

The main findings of this work can be resumed summarized as follows: (i) in both STABLE and VULN groups, autonomic markers did not vary with orthostatic challenge, thus suggesting that the autonomic control is impaired; (ii) VULN patients had a lower YM; (iii) the VULN group’s BRS_HF_ decreased during STAND compared to REST, being higher at REST compared to the STABLE group; (iv) elastographic markers were significantly correlated with autonomic variability in VULN patients but not in the STABLE ones; (v) the joint analysis of Young’s modulus and baroreflex markers improved the predictability of the plaque vulnerability.

### 4.1. Autonomic Control Is Impaired in Both Patients with Vulnerable and Stable Plaque

The assessment of autonomic function markers in patients with asymptomatic CAS allowed the detection of an autonomic impairment before the intervention, regardless of the kind of carotid plaque [25,26]. In fact, both STABLE and VULN populations did not present the expected response to the postural challenge in terms of a decrease of HF_HP_ and increase of LF_SAP_ [35,36,37]. The missing response to STAND confirmed the state of sympathetic activation and vagal withdrawal already suggested in patients featuring CAS [25,26]. Remarkably, plaque removal does not assure any improvement in the autonomic control likely because the surgical procedure could damage sensing areas of the carotid artery, with erroneous perception of the baroreflex loading [38,39].

### 4.2. Baroreflex Function at the Respiratory Rate Is Preserved in Patients with Vulnerable Plaque

One of the most interesting findings of this study is that at REST the BRS_HF_ was higher in the VULN group compared to the STABLE one, and it decreased, as expected [29,36], during STAND. This finding suggests that the baroreflex function could be more preserved in presence of a VULN plaque with respect to a STABLE one, since this population showed a better ability of modulating the baroreflex in response to the postural challenge compared to the STABLE group. Given this result, we suggest that subjects with STABLE plaque might be exposed to greater cardiovascular risk because they are less effective in compensating changes in SAP with suitable adaptations of HP, compared to VULN patients [18]. The greater stiffness of the substrate of the barosensitive areas where the mechanoreceptors are located might be responsible for the lower values of BRS_HF_ observed in the STABLE group compared to the VULN one. However, this speculation lacks appropriate direct evidence. Remarkably, at difference with the BRS_HF_, the BRS_LF_ in the VULN group did not change with STAND. This finding might appear to be surprising given that BRS_LF_ is more reliable than BRS_HF_ in probing baroreflex function [36,40]. This greater statistical power of BRS_HF_ compared to BRS_LF_ in separating experimental conditions and groups might again support the concept that the observed impairment of baroreflex control in the STABLE group is more related to the mechanical transduction than the neural arch of cardiac baroreflex. Indeed, BRS_HF_ is more related to the ability of modifications of intrathoracic pressure during respiration [41] to solicit the site where the mechanoreceptors are located, being stiffer areas less able to sense modifications of arterial pressure.

### 4.3. The Relationship Between Elastographic and Autonomic Markers Is Different in Patients with Vulnerable or Stable Plaque

A preliminary study performed in a smaller population [17] allowed us to determine that patients with a vulnerable plaque could be differentiated from those with a stable one thanks to the assessment of elastographic markers and especially to the YM of the plaque, being lower in the VULN patients than in the STABLE ones. This work presents the same parameters in a larger population, confirming previous findings. In fact, YM was again the only marker able to differentiate populations, while the other markers related to arterial stiffness were not different between the two populations, being confirmed as a prognostic descriptor of the plaque’s vulnerability. This ability of YM could be explained by the fact that the formation of the atherosclerotic plaque could lead to a change in the biomechanics of the vessel [42,43,44].

This study originally also presented a correlation analysis between elastographic and autonomic markers, performed separately in VULN and STABLE patients. In STABLE patients, we did not observe significant correlations, with the sole exception of an index of baroreflex function such as α_LF_ and index of arterial stiffness such as AIX. On the contrary, in VULN patients, we found several significant correlations between elastographic and autonomic markers. In this group, the higher variability of markers of sympathetic modulation, such as σ^2^_SAP_ and more specifically LF_SAP_, might have favored the detection of a significant positive association with the YM of the plaque. This finding would suggest that patients with a more elastic plaque would be prone to having more variable and reactive cardiovascular control.

Furthermore, we found that in the VULN group, the higher the parasympathetic modulation directed to the heart during STAND, represented by HF_HP_, the higher the stiffness of the carotid artery, represented by α, β index, and PWV. We suggest that within the group with the less remarkable value of stiffness, i.e., the VULN group, further differentiation can be carried out in terms of the response of the autonomic control to compensate greater stiffness values. In the STABLE group, any correlation is lost likely because the level of stiffness of the vasculature reached by this group prevents any correlation. Anyway, these findings can be considered only at a speculative level since, to our knowledge, a study exploring the association of elastographic and cardiovascular control markers has not been performed so far and should be confirmed in a control group, age and gender matched with our population.

### 4.4. The Joint Analysis of Autonomic and Elastographic Markers Improves the Predictability of Plaque Vulnerability

This work originally assessed the ability of both elastographic markers and cardiovascular control indexes to predict the vulnerability of the plaque via a logistic regression analysis. With respect to preliminary findings, this work assessed the ability of different elastographic markers to predict the vulnerability of the plaque. Anyway, only YM was found to be associated with plaque’s vulnerability, with an AUC of 0.625 when used alone to predict the outcome. All the autonomic and cardiovascular control markers assessed at REST and STAND were tested for association with the plaque’s vulnerability. Again, the marker able to discriminate VULN and STABLE with a probability of type-I error equal or lower to 0.1 was BRS_HF_ evaluated during REST, which was then tested first at the univariate level, entering a logistic regression analysis, reporting an AUC of 0.616. Remarkably, the multivariate logistic regression model built starting from the two markers reported a higher AUC, being equal to 0.694, further improving the results also in terms of positive and negative predictive value. These findings showed that the YM was the most powerful marker to discriminate plaque’s vulnerability, but the addition of baroreflex analysis was shown to be able to increase the prediction ability.

Remarkably, since the performed analyses were noninvasive and preoperative, we suggest that they could be used in standard clinical practice to better determine the risk profile of the patients with asymptomatic CAS, thus helping clinicians in defining the better treatment for CAS patients by admitting to surgery only those with a higher risk of rupture of the plaque and, consequently, of stroke.

### 4.5. Limitations and Future Developments

We remark that this study did not consider comparisons with a proper control group, either made of healthy subjects or of symptomatic patients. It would be desirable in the future to provide reference values useful to scale the markers reported in this study. Furthermore, the presence of beta-blockers or other medications should also be considered in the model as confounding factors, given their ability to influence autonomic control. Novel therapies, in particular sodium-glucose co-transporter 2 (SGLT2) inhibitors, have been recently introduced, showing an efficacy to reduce vascular stiffness [45]. Future studies could be aimed at testing whether they have a synergistic effect with the markers proposed in this study. Furthermore, future studies will be depicted to enlarge the population and to test more advanced signal processing techniques to determine if the prediction can be further improved. While confirmed, joint baroreflex and elastographic analysis to predict the vulnerability of the plaque could help in improving risk stratification in CAS patients and in tailoring therapies according to the risk of developing stroke.

## 5. Conclusions

This work originally presented a joint analysis of shear wave elastography, together with an RF-echo-based wall tracking method for arterial stiffness evaluation, autonomic function, and baroreflex control in CAS patients, to determine differences between those with STABLE and VULN carotid plaque. Remarkably, patients with VULN plaque, considered to be at higher clinical risk, presented a lower YM of the plaque and a preserved baroreflex response to the orthostatic challenge. Furthermore, as reported by a correlation analysis, a relation between elastographic and cardiovascular control markers was present only in this group. This work suggests that elastographic indexes are useful to predict the vulnerability of the plaque and that the addition of BRS indexes further improves the prediction, thus confirming the clinical value of baroreflex markers especially in asymptomatic CAS patients, as well as strengthening the validity of elastographic techniques.

## Figures and Tables

**Table 1:** Demographic and clinical parameters in STABLE and VULN patients.

Parameter	STABLE (*n* = 42)	VULN (*n* = 36)
age [yrs]	74.93 ± 7.30	73.39 ± 8.31
gender [female]	19 (45)	10 (28)
BMI [kg·m^−2^]	25.96 ± 3.85	25.73 ± 4.20
hypertension	39 (93)	30 (83)
beta-blockers	13 (31)	13 (36)
diabetes	14 (33)	12 (33)
smoke	10 (24)	7 (19)
previous smoke	19 (45)	18 (50)
dyslipidemia	39 (93)	30 (83)
CAD	8 (19)	9 (25)
COPD	2 (5)	0 (0)
previous cerebrovascular events	3 (7)	6 (8)
% of stenosis	80.00 ± 4.42	80.28 ± 4.77

BMI = body mass index; CAD = coronary artery disease; COPD= chronic obstructive pulmonary disease. Continuous data are presented as mean ± standard deviation and categorical data as number (percentage).

**Table 2:** Elastographic markers from QAS and pSWE in STABLE and VULN patients.

Index	STABLE	VULN
DC [1·kPa^−1^]	0.010 ± 0.007	0.010 ± 0.006
CC [mm^2^·kPa^−1^]	0.595 ± 0.36	0.611 ± 0.335
α stiffness	11.239 ± 11.201	9.581 ± 5.188
β stiffness	22.742 ± 22.438	19.426 ± 10.4
PWV [m·s^−1^]	11.385 ± 5.105	11.058 ± 3.139
AIx [%]	8.195 ± 8.639	6.241 ± 5.412
YM [kPa]	46.211 ± 45.384	25.653 ± 27.237 *

DC = distensibility coefficient; CC = compliance coefficient; α stiffness = elastic coefficient of the vessel; β stiffness = elastic coefficient normalized on the diameter; PWV = pulse wave velocity; AIx = augmentation index; YM = Young’s modulus of the plaque. STABLE = patients with a stable plaque; VULN = patients with a vulnerable plaque. The symbol * means *p* < 0.05 versus STABLE.

**Table 3:** Autonomic and cardiovascular control markers in STABLE and VULN patients during REST and STAND.

Index	STABLE		VULN	
	REST	STAND	REST	STAND
µ_HP_ [ms]	882.92 ± 137.41	824.36 ± 185.92	924.75 ± 170.57	846.32 ± 227.07 *
σ^2^_HP_ [ms^2^]	581.86 ± 568.46	509.99 ± 506.19	687.05 ± 617.74	660.14 ± 639.33
HF_HP_ [ms^2^]	153.98 ± 264.35	110.8 ± 161.11	157.97 ± 191.69	128.14 ± 179.63
LF_HP_/HF_HP_	1.36 ± 1.86	1.46 ± 1.64	6.77 ± 34.74	1.59 ± 1.84
f_RESP_ [Hz]	0.27 ± 0.04	0.27 ± 0.06	0.27 ± 0.04	0.28 ± 0.06
µ_SAP_ [mmHg]	147.44 ± 18.79	150.66 ± 29.94	144.35 ± 17.93	144.19 ± 31.34
σ^2^_SAP_ [mmHg^2^]	35.29 ± 30.43	35.49 ± 25.94	31.38 ± 32.82	36.04 ± 27.03
LF_SAP_ [mmHg^2^]	4.35 ± 4.3	5.56 ± 7.53	4.06 ± 4.23	7.32 ± 10.41
α_LF_ [ms∙mmHg^−1^]	5.34 ± 4.64	5.46 ± 6.32	7.67 ± 9.35	5.29 ± 5.56
α_HF_ [ms∙mmHg^−1^]	4.97 ± 4.29	4.53 ± 3.74	6.91 ± 6.16	5.33 ± 5.38
K^2^_LF_	0.34 ± 0.18	0.33 ± 0.17	0.31 ± 0.19	0.36 ± 0.17
Ph_LF_ [rad]	−1.15 ± 1.37	−1.4 ± 1.21	−1.42 ± 1.22	−1.1 ± 1.28
BRS_LF_ [ms∙mmHg^−1^]	2.69 ± 1.98	2.35 ± 1.71	3.34 ± 2.85	2.64 ± 1.96
K^2^_HF_	0.63 ± 0.22	0.62 ± 0.27	0.62 ± 0.26	0.55 ± 0.26
Ph_HF_ [rad]	−0.32 ± 0.87	−0.25 ± 1.12	−0.26 ± 1.01	0.02 ± 0.98
BRS_HF_ [ms∙mmHg^−1^]	4.01 ± 4.12	3.42 ± 3.15	6.16 ± 6.19 §	3.81 ± 3.66 *

REST = at rest in supine position; STAND = during active standing; VULN = patients with vulnerable carotid plaque; STABLE = patients with stable carotid plaque; HP = heart period; SAP = systolic arterial pressure; μ = mean; σ^2^ = variance; LF = low frequency; HF = high frequency. The symbol * indicates *p* < 0.05 with respect to REST. The symbol § indicates *p* < 0.05 with respect to STABLE.

**Table 4:** Pearson’s correlation coefficient r between elastographic and cardiovascular control markers in STABLE patients during REST and STAND.

Index	DC [1·kPa^−1^]	CC [mm^2^·kPa^−1^]	α index	β index	PWV [m·s^−1^]	AIx [%]	YM [kPa]
µ_HP_ REST [ms]	0.120	0.178	−0.217	−0.215	−0.257	−0.056	−0.007
σ^2^_HP_ REST [ms^2^]	−0.032	−0.040	−0.072	−0.072	−0.039	0.211	0.043
HF_HP_ REST [ms^2^]	−0.030	−0.153	−0.108	−0.108	−0.026	0.153	−0.020
LF_RR_/HF_RR_ REST	0.285	0.325	−0.160	−0.161	−0.209	−0.253	0.076
f_RESP_ REST [Hz]	0.0642	0.00808	0.2	0.0653	0.00353	0.00379	0.036
µ_SAP_ REST [mmHg]	−0.193	−0.256	0.066	0.068	0.172	0.148	−0.130
σ^2^_SAP_ REST [mmHg^2^]	0.143	0.193	−0.039	−0.041	−0.107	0.060	0.212
LF_SAP_ REST [mmHg^2^]	−0.111	−0.035	0.170	0.169	0.138	−0.106	0.243
α_LF_ REST [ms∙mmHg^−1^]	−0.228	−0.211	0.084	0.084	0.173	0.349 *	0.076
α_HF_ REST [ms∙mmHg^−1^]	0.035	−0.099	−0.115	−0.114	−0.068	−0.052	0.017
K^2^_LF_ REST	0.151	−0.022	−0.085	−0.085	−0.111	−0.235	−0.089
Ph_LF_ REST [rad]	−0.245	−0.031	0.128	0.129	0.133	0.256	0.237
BRS_LF_ REST [ms∙mmHg^−1^]	−0.198	−0.221	0.078	0.079	0.139	0.264	0.142
K^2^_HF_ REST	0.302	0.095	−0.152	−0.152	−0.170	−0.274	−0.239
Ph_HF_ REST [rad]	0.276	0.171	−0.242	−0.240	−0.268	−0.248	−0.210
BRS_HF_ REST [ms∙mmHg^−1^]	0.139	0.097	−0.185	−0.184	−0.135	−0.140	−0.158
µ_HP_ STAND [ms]	0.011	0.093	−0.097	−0.094	−0.124	−0.003	0.056
σ^2^_HP_ STAND [ms^2^]	0.020	0.035	−0.083	−0.083	−0.082	−0.166	−0.028
HF_HP_ STAND [ms^2^]	0.027	−0.158	−0.092	−0.092	−0.064	−0.166	−0.054
LF_HP_/HF_HP_ STAND	−0.065	0.024	0.011	0.011	−0.009	0.207	−0.018
f_RESP_ STAND [Hz]	−0.0642	−0.0317	0.0125	−0.102	−0.118	−0.119	−0.0567
µ_SAP_ STAND [mmHg]	−0.246	−0.270	0.122	0.124	0.244	0.134	−0.061
σ^2^_SAP_ STAND [mmHg^2^]	0.091	0.309	−0.178	−0.178	−0.220	0.053	−0.155
LF_SAP_ STAND [mmHg^2^]	0.014	0.086	−0.149	−0.149	−0.087	0.048	−0.088
α_LF_ STAND [ms∙mmHg^−1^]	−0.123	−0.269	0.146	0.145	0.127	−0.064	0.252
α_HF_ STAND [ms∙mmHg^−1^]	0.001	−0.121	−0.019	−0.020	−0.006	−0.196	0.103
K^2^_LF_ STAND	0.124	0.063	0.049	0.048	−0.017	−0.033	−0.031
Ph_LF_ STAND [rad]	−0.204	−0.132	0.185	0.186	0.126	−0.091	0.235
BRS_LF_ STAND [ms∙mmHg^−1^]	−0.167	−0.214	0.228	0.228	0.187	−0.016	0.235
K^2^_HF_ STAND	0.164	−0.081	−0.079	−0.080	−0.009	−0.046	0.011
Ph_HF_ STAND [rad]	0.123	0.051	−0.167	−0.164	−0.151	0.122	−0.093
BRS_HF_ STAND [ms∙mmHg^−1^]	−0.032	−0.069	0.028	0.027	0.057	−0.163	0.130

REST = at rest in supine position; STAND = during active standing; DC = distensibility coefficient; CC = compliance coefficient; α stiffness = stiffness, elastic coefficient of the vessel; β stiffness = elastic coefficient normalized on the diameter; PWV = pulse wave velocity; AIx = augmentation index; YM = Young’s modulus of the plaque; HP = heart period; SAP = systolic arterial pressure; μ = mean; σ^2^ = variance; LF = low frequency; HF = high frequency. The symbol * indicates significant correlation with *p* < 0.05.

**Table 5:** Pearson’s correlation coefficient r between elastographic and cardiovascular control markers in VULN patients during REST and STAND.

Index	DC [1·kPa^−1^]	CC [mm^2^·kPa^−1^]	α index	β index	PWV [m·s^−1^]	AIx [%]	YM [kPa]
µ_HP_ REST [ms]	−0.241	−0.133	0.108	0.108	0.179	−0.210	0.457 *
σ^2^_HP_ REST [ms^2^]	−0.051	0.122	0.111	0.111	0.076	0.100	0.082
HF_HP_ REST [ms^2^]	−0.114	−0.005	0.176	0.176	0.121	0.003	0.178
LF_HP_/HF_HP_ REST	0.063	0.067	−0.127	−0.128	−0.089	−0.069	0.073
f_RESP_ REST [Hz]	−0.282	0.044	−0.14	−0.18	0.174	0.173	0.24
µ_SAP_ REST [mmHg]	−0.035	0.068	−0.077	−0.077	0.018	0.235	0.290
σ^2^_SAP_ REST [mmHg^2^]	−0.309 *	−0.255	0.190	0.191	0.312 *	−0.048	0.190
LF_SAP_ REST [mmHg^2^]	−0.155	−0.150	0.090	0.091	0.180	−0.104	0.37 *
α_LF_ REST [ms∙mmHg^−1^]	−0.021	0.093	−0.010	−0.010	−0.040	−0.075	−0.036
α_HF_ REST [ms∙mmHg^−1^]	−0.006	0.047	−0.012	−0.012	−0.058	−0.090	−0.068
K^2^_LF_ REST	0.100	−0.001	−0.180	−0.180	−0.240	−0.092	−0.179
Ph_LF_ REST [rad]	−0.143	−0.201	0.163	0.163	0.208	0.484 *	−0.011
BRS_LF_ REST [ms∙mmHg^−1^]	0.105	0.109	−0.092	−0.093	−0.167	−0.076	−0.084
K^2^_HF_ REST	−0.110	−0.214	−0.008	−0.008	−0.035	0.171	−0.133
Ph_HF_ REST [rad]	0.175	0.152	0.077	0.077	−0.062	−0.026	−0.287
BRS_HF_ REST [ms∙mmHg^−1^]	−0.058	0.003	−0.046	−0.046	−0.056	−0.159	−0.095
µ_HP_ STAND [ms]	−0.357 *	−0.258	0.152	0.153	0.274	−0.188	0.412 *
σ^2^_HP_ STAND [ms^2^]	−0.175	−0.017	0.219	0.219	0.258	0.050	0.095
HF_HP_ STAND [ms^2^]	−0.289	−0.187	0.424 *	0.425 *	0.443 *	0.027	0.193
LF_HP_/HF_HP_ STAND	0.176	0.169	−0.199	−0.199	−0.198	−0.053	−0.061
f_RESP_ STAND [Hz]	−0.0882	0.165	−0.201	−0.117	0.198	0.199	0.27
µ_SAP_ STAND [mmHg]	−0.325 *	−0.345 *	0.096	0.097	0.117	−0.097	0.361 *
σ^2^_SAP_ STAND [mmHg^2^]	−0.086	−0.071	−0.069	−0.069	−0.185	−0.157	0.328 *
LF_SAP_ STAND [mmHg^2^]	−0.068	−0.046	−0.057	−0.056	−0.033	−0.147	0.130
α_LF_ STAND [ms∙mmHg^−1^]	−0.195	−0.122	0.119	0.120	0.149	0.185	−0.117
α_HF_ STAND [ms∙mmHg^−1^]	−0.160	−0.032	0.300	0.300	0.295	0.167	−0.116
K^2^_LF_ STAND	0.119	0.031	−0.236	−0.236	−0.157	0.153	−0.065
Ph_LF_ STAND [rad]	−0.167	−0.103	−0.005	−0.004	0.133	−0.156	0.037
BRS_LF_ STAND [ms∙mmHg^−1^]	−0.035	0.127	−0.057	−0.057	−0.002	0.129	−0.159
K^2^_HF_ STAND	0.126	0.096	−0.192	−0.193	−0.174	0.232	0.154
Ph_HF_ STAND [rad]	0.244	0.137	−0.121	−0.121	−0.264	0.083	0.013
BRS_HF_ STAND [ms∙mmHg^−1^]	−0.109	0.048	0.088	0.088	0.108	0.001	−0.083

REST = at rest in supine position; STAND = during active standing; DC = distensibility coefficient; CC = compliance coefficient; α stiffness = stiffness, elastic coefficient of the vessel; β stiffness = elastic coefficient normalized on the diameter; PWV = pulse wave velocity; AIx = augmentation index; YM = Young’s modulus of the plaque; HP = heart period; SAP = systolic arterial pressure; μ = mean; σ^2^ = variance; LF = low frequency; HF = high frequency. The symbol * indicates significant correlation with *p* < 0.05.

**Table 6:** Results of multivariate logistic regression analysis for plaque’s vulnerability prediction.

Parameter	Regression Coefficient	Odds Ratio	95% CI	*p*-Value	AUC
YM	−0.014	0.986	0.973–1.000	0.043	0.694
BRS_HF_ REST	0.075	1.078	0.973–1.194	0.153	
constant	−0.038	0.963		0.928	

YM = Young’s modulus of the plaque. BRS = baroreflex sensitivity; HF = high frequency; BRS_HF_ = BRS in HF band evaluated at REST; CI = confidence interval; AUC = area under the ROC curve.

## Data Availability

Data are available upon reasonable request to the corresponding author.
